# An improved quantitative real-time polymerase chain reaction technology for *Helicobacter pylori* detection in stomach tissue and its application value in clinical precision testing

**DOI:** 10.1186/s12896-020-00624-z

**Published:** 2020-06-22

**Authors:** Ling Deng, Xiao-Yi He, Bin Tang, Yang Xiang, Juan-Juan Yue

**Affiliations:** 1 Department of Clinical Microbiology and Immunology, Faculty of Pharmacy and Medical Laboratory Sciences, Third Military Medical University (Army Medical University), No. 30 Gaotanyan Street, Chongqing, 400038 People’s Republic of China; 2 Department of Digestive Disease Center, The Third Affiliated Hospital of Chongqing Medical University (General Hospital), Chongqing, China

**Keywords:** Quantitative detection, Precision testing, *Helicobacter pylori*

## Abstract

**Background:**

*Helicobacter pylori* (*H. pylori*) infection is a serious human health threat. The empiric *H. pylori* treatment paradigm guided by traditional testing technologies has led to antibiotic resistance. Here, we improved the qPCR method to provide technical support for precision *H. pylori* diagnosis and treatment.

**Methods:**

Two pairs of primers and probes targeting the glmM gene were designed to detect *H. pylori*, and a multiplex qPCR method was established for virulence factor detection. Then, a rapid urease test (RUT), culturing and qPCR were performed on 141 specimens collected from Xinqiao Hospital of China in 2017 to evaluate the qPCR detection capability. Finally, the *H. pylori* infectious amount and virulence genes were detected by qPCR.

**Results:**

1. The improved qPCR method which used two pairs of primers had a higher detection rate (100%) and better accuracy (*p* = 0.000), compared with the qPCR using a pair of primers. It also had better consistency with the bacterial culture than with RUT (Kappa =0.440, *p* < 0.001). 2. The *H. pylori* infectious amount was significantly positively associated with gastritis in corpus (*p* = 0.003) and gastric erosion (*p* = 0.043). The *H. pylori* infectious amount in gastric precancerous patients was significantly lower than that in *H. pylori*-positive patients (*p* < 0.05), and the infectious *H. pylori*-vacA s1+ amount was significantly greater than that of *H. pylori*-vacA s1- (p < 0.05). 3. The vacA s1 frequency was significantly higher than that of vacA m1/cagA+/babA2+ in chronic superficial gastritis (*p* = 0.000), peptic ulcer (*p* = 0.037) and gastric erosion (*p* = 0.009). The *H. pylori*-vacA+/cagA+/babA2+ frequency showed a significant positive correlation (*p* < 0.05).

**Conclusions:**

The *H. pylori* infectious amount and presence of *H. pylori* virulence factors showed complex correlations with gastric disease occurrence and development. The improved qPCR with good detection performance can be used for quantitative *H. pylori* detection and testing for the virulence genes vacA s1, vacA m1, cagA and babA2 simultaneously. These findings will provide valuable information for disease diagnosis and treatment.

## Background

*Helicobacter pylori* (*H. pylori*) is a bacterial pathogen that infects more than half of the world’s population [1]. The infection is associated with gastrointestinal symptomatology, from gastritis to gastric cancer. In 1994, the World Health Organization (WHO) International Agency for Research on Cancer (IARC) classified *H. pylori* as a group I carcinogen [2]. *H. pylori* eradication has now been recommended as the primary strategy for preventing gastric cancer in all recently developed guidelines [3–5]. However, empiric drug use under the guidance of traditional detection technology has caused many problems, such as antibiotic resistance, which is becoming increasingly serious [6–8]. In 2019, Barry Marshall suggested that the empiric *H. pylori* treatment paradigm should be changed to test-guided precision treatment [9]. The transformation of the treatment model is based on the development of precision testing technology.

It is necessary to point out that not all carriers develop severe gastrointestinal diseases with clinical symptoms. The genomes of *H. pylori* are heterogeneous and encode different virulence factors that play an important role in the clinical outcome of the infection. Among them, the proteins encoded by the vacA, cagA and babA2 genes determine the pathogenicity of *H. pylori* and have been well described [10].

Vacuolating cytotoxin A (VacA) is a multifunctional toxin. VacA activities, in addition to vacuolation, include interference in the mitochondrial membrane, the stimulation of apoptosis, the blocking of T cell proliferation and so on, which assists *H. pylori* in colonizing stomach epithelial cells in humans [11, 12]. The vacA gene has variable structures in the signal region(s), with s1 or s2 allele types, and the intermediate region (i), which has subtypes 1 and 2, while the middle region (m) has m1 and m2 allele types. The vacA s1/i1/m1 allele is thought to secrete an active VacA, which mediates an increased chance of peptic ulceration and leads to the development of gastric cancer [12–14]. Cytotoxin-associated gene A (CagA) is an effector protein encoded by the cagA gene. The cagA gene is localized within a 40-kb chromosomal region known as the cag pathogenicity island (cag-PAI) that consists of 27–31 genes, including those that encode a type IV secretion system (T4SS), which is responsible for the translocation of CagA to the cytoplasm of gastric epithelial cells in cagA-positive strains of *H. pylori* [15]. *H. pylori* CagA plays a critical role in gastric inflammation and is associated with a significantly increased risk for the development of ulcer disease or gastric cancer [16, 17]. Another important *H. pylori* virulence factor is the blood group antigen-binding adhesin (BabA), which is one of the best-characterized adhesion proteins of the bacterium. It binds to the fucosylated Lewis B antigen (Le b) present on the surface of gastric epithelial cells and facilitates the colonization, persistence of infection and virulence factor release of the bacterium [18]. BabA protein is encoded by the genes babA1 and babA2. Only babA2 encodes the functionally active protein, which is required for the binding of *H. pylori* to Lewis B [19]. Infection with babA2-positive *H. pylori* has been associated with gastric adenocarcinoma, gastric ulcer and duodenal ulcer. The risk of severe disease will increase when it coexists with the cagA gene and the vacA s1 allele [18, 20].

Because of its serious harm to human health, the clinical diagnosis *H. pylori* is particularly important. Traditional diagnostic tests are usually divided into invasive and noninvasive methods. Invasive diagnostic tests include endoscopic imaging, histology, a rapid urease test, culture and molecular methods. Noninvasive diagnostic tests included a urea breath test, a stool antigen test and so on [21]. Polymerase chain reaction (PCR) is a molecular method that has been used extensively for the detection of *H. pylori* infection and the evaluation of the virulence factors and antibiotic sensitivity of *H. pylori* [22, 23]. It has several advantages, such as excellent sensitivity and specificity, fast results and no need for special processing supplies or transportation [21]. However, in contrast to the rapid and highly accurate results obtained by PCR for the detection of *H. pylori* infection and antibiotic-resistant strains, concerns about cost, equipment availability and expertise in molecular techniques inevitably influence the feasibility of PCR in local laboratories and clinical practice [21, 22].

The objective of this research was to improve the quantitative real-time polymerase chain reaction (qPCR) method by designing two pairs of primers and probes targeting the glmM gene to quantitatively detect *H. pylori* and identify for the virulence genes vacA s1, vacA m1, cagA and babA2. We also used the method to test for *H. pylori* in patients and analysed the effective information within the data. The method showed a promising clinical application prospect and may be capable of providing a precise clinical diagnosis of *H. pylori*.

## Results

### Performance of qPCR

A series of 10-fold dilutions of plasmid DNA (ranging from 1 × 10^4^ to 1 × 10^9^ copies/μL) were used as the template and tested in quantitative RT-PCRs, all in triplicate.

**Fig. 1:**
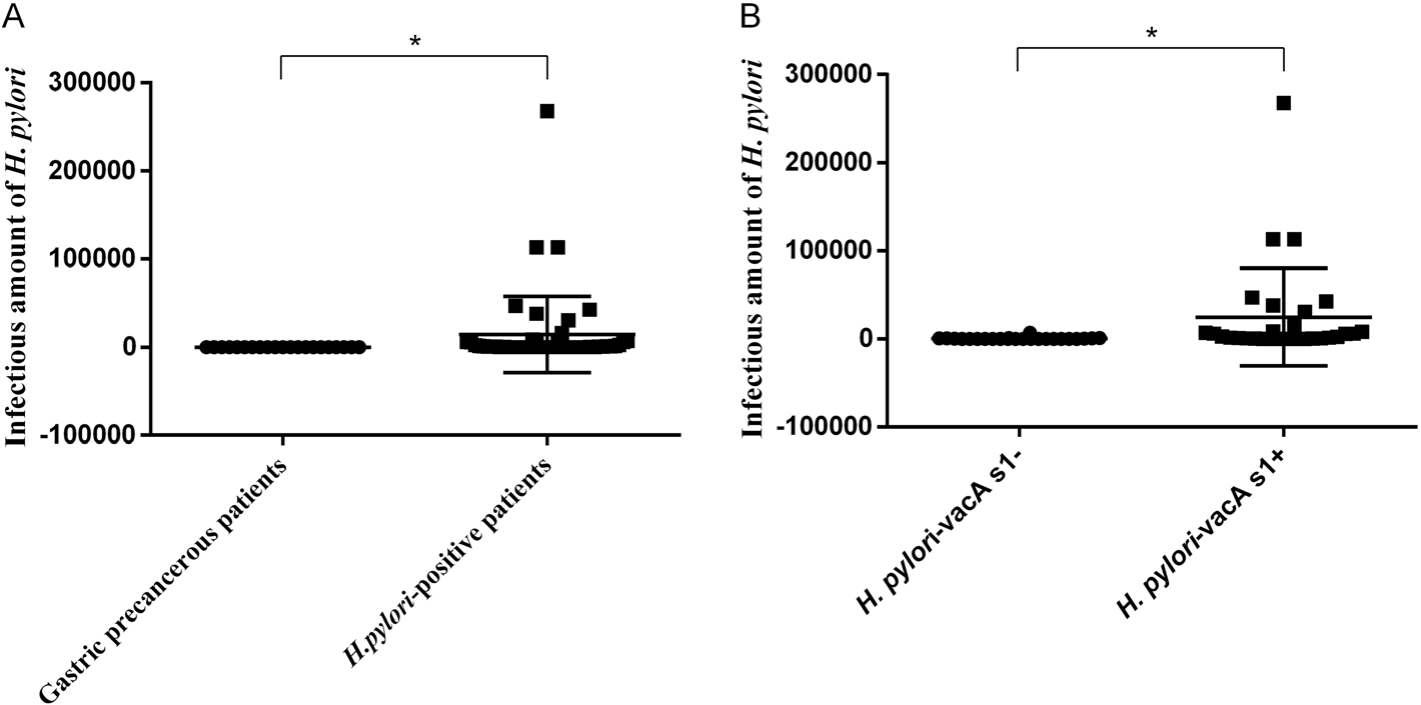
Comparison of the number of *H. pylori* copies between different groups. **a.** Gastric precancerous patients and other *H. pylori*-positive patients. **b.** The vacA s1- and the vacA s1+ groups. * *p* < 0.05

The detection rate of two pairs of primers (100%) was higher than that of a pair of primers (15%). Meanwhile, the mean copy number of the method with a pair of primers (4.10 ± 1.09) was lower than those of the method with two pairs of primers (6.57 ± 0.49). The difference was significant (t = − 9.309, *p* = 0.000) (Supplementary Table 1 and Figure 1). The limit of detection (LOD) of glmM was 100 colony-forming units (CFU), with an average Ct value of 34.58, which is equivalent to 6 bacterial copies. The area under the ROC curve (AUC) was 0.988, and the Youden index was 0.98. The LODs of vacA s1, vacA m1, cagA and babA2 were 200 CFU, 100 CFU, 200 CFU and 100 CFU, respectively. The AUC values were 0.986, 0.988, 0.987 and 0.984, and the Youden index values were 0.94, 0.95, 0.9 and 0.9, respectively.

The specificity, sensitivity and positive and negative predictive values (PPVs and NPVs, respectively) of real-time PCR are given in Supplementary Table 2 for all 134 biopsy specimens. In our study, this real-time PCR assay showed a sensitivity of 100% (NPV, 100%) and a specificity of 70.5% (PPV, 40%).

### Determination of *H. pylori* infection status

By using the criteria adopted for this study, 53 of the 134 (39.6%) tested patients were positive for *H. pylori* (Supplementary Table 3), and 7 patients were excluded because of only one test result. Of these patients, 20 gave concordant positive results (group I) by all three methods (RUT, qPCR and culturing of *H. pylori*). For another 2 patients (groups II and III), the RUT was not performed or gave negative results, and for another 31 patients (group IV), the culturing gave negative results. However, *H. pylori* was concordantly identified by the remaining two methods.

### Comparison of the three methods

The positive detection rates of the three methods (RUT, qPCR and culture) were 84.3, 41 and 16.4%, respectively. Compared with the RUT method, qPCR had better consistency with the classic gold standard of bacterial culture (Kappa =0.440, *p* < 0.001) (Table 1).

**Table 1:** Consistency comparison of qPCR, RUT and culturing

		Culture		Kappa	P (McNemar)
		Positive	Negative		
**qPCR**	**Positive**	22	33	0.440	0.000
	**Negative**	0	79		
**RUT**	**Positive**	20	87	0.050	0.000
	**Negative**	1	19		

### The quantitative detection of *H. pylori*

The amount of *H. pylori* in gastric precancerous patients (13.12 ± 26.56) was significantly lower than that in other *H. pylori*-positive patients (14,478.11 ± 43,307.22) (Fig. 1a), and the *H. pylori*-vacA s1+ infectious amount (24,985.20 ± 55,518.99) was significantly higher than the *H. pylori*-vacA s1- infectious amount (580.83 ± 1451.70) (Fig. 1b). The difference was significant (*p* < 0.05). There was no significant difference in copy numbers among different ages, genders, gastric diseases and genotype strains in *H. pylori*-positive infection patients(*n* = 53) (*P* > 0.05) (Table 2). We also analysed the 134 patients and found that the infectious amount of *H. pylori* was significantly positively associated with gastritis in corpus (r = 0.259, *p* = 0.003) and gastric erosion (r = 0.175, *p* = 0.043).

**Table 2:** Comparison of *H. pylori* infection among different variables (*n* = 53)

Variety	Group	n	Mean ± sd	t	p
Age	≤50	28	20,031.05 ± 54,049.41	1.106	0.274
	> 50	25	7105.65 ± 23,356.77		
Gender	Male	25	16,632.10 ± 53,889.88	0.433	0.667
	Female	28	11,525.30 ± 29,896.31		
Gastric disease	CSG	45	15,287.95 ± 45,840.75	0.546	0.588
	CAG	8	6319.10 ± 12,921.64		
Genotype	*H. pylori*-vacA m1+	7	15,624.29 ± 18,803.06	0.112	0.911
	*H. pylori*-vacA m1-	46	13,676.97 ± 45,223.21		
	*H. pylori*-cagA+	14	21,849.63 ± 39,784.22	0.809	0.423
	*H. pylori*-cagA-	39	11,092.72 ± 43,651.55		
	*H. pylori*-babA2+	14	24,080.55 ± 39,472.15	1.041	0.303
	*H. pylori*-babA2-	39	10,291.87 ± 43,517.17		

### H. Pylori virulence factor detection

We found that 58.5% (31/53) of the patients were infected with *H. pylori* containing virulence factors (Supplementary Table 4). The vacA s1 genotype was the most frequent among *H. pylori*-positive patients, with 54.7% (29/53). Among these, m1 was found in co-infection with s1 in 13.2% of patients (7/53). In 41.5% (22/53) of the patients, the s1 allele was detected, but the m1 region was undetectable. Fourteen of the 53 *H. pylori*-positive biopsies were tested for cagA. The babA2 gene was detected in 26.4% (14/53) of *H. pylori*-positive patients. There were no significant differences in the frequency of *H. pylori*-vacA+/cagA+/babA2+ strains among different diseases. However, significant differences were found in the frequency of virulence factors in the same disease (Table 3). Among these variations, the frequency of vacA s1 was significantly higher than that of vacA m1/cagA+/babA2+ in chronic superficial gastritis (CSG)(*p* = 0.000), peptic ulcer(*p* = 0.037) and gastric erosion (*p* = 0.009).

**Table 3:** The status of the vacA, cagA and babA2 genotypes in patients with gastric diseases

	Diagnosis				*p* value (x^2^test)
	CSG n(%)	CAG n(%)	Peptic ulcer n(%)	Gastric erosion n(%)	
*H. pylori*					
Negative	72 (53.7)	9 (6.7)	11 (8.2)	26 (19.4)	0.509
Positive	45 (33.6)	8 (6)	10 (7.5)	26 (19.4)	
vacA s1					
Negative	21 (46.7)	3 (37.5)	2 (20)	12 (46.2)	0.456
Positive	24 (53.3)	5 (62.5)	8 (80)	14 (53.8)	
vacA m1					
Negative	40 (88.9)	6 (75)	8 (80)	23 (88.5)	0.665
Positive	5 (11.1)	2 (25)	2 (20)	3 (11.5)	
cagA					
Negative	35 (77.8)	4 (50)	7 (70)	19 (73.1)	0.414
Positive	10 (22.2)	4 (50)	3 (30)	7 (26.9)	
babA2					
Negative	32 (71.1)	7 (87.5)	6 (60)	19 (73.1)	0.638
Positive	13 (28.9)	1 (12.5)	4 (40)	7 (26.9)	
p value (x^2^ test)	0.000	0.149	0.037	0.009	

The frequency of *H. pylori*-vacA+/cagA+/babA2+ strains showed a significant positive correlation (Table 4). Among these strains, the babA2 genotype had a higher degree of association with vacA s1(r = 0.459, *p* = 0.001) and cagA (r = 0.418, *p* = 0.002) than with vacA m1 (r = 0.272, *p* = 0.049).

**Table 4:** The correlations between the survival rates of *H. pylori* and the virulence factors

	vacA s1	vacA m1	cagA	babA2
vacA s1	1.000			
vacA m1	0.355^**^	1.000		
cagA	0.459^**^	0.398^**^	1.000	
babA2	0.459^**^	0.272^*^	0.418^**^	1.000

**p < 0.01, * p < 0.05

## Discussion

Quantitative RT-PCR is a molecular method of detecting *H. pylori* that has been recently rapidly developed. Compared with conventional PCR, real-time PCR has several advantages, such as short working time, high specificity, and low risk of contamination. In the past, several target genes, including ureA, glmM, ureC, 16SrRNA, 23SrRNA, hsp60 and vacA, had been used for the detection of *H. pylori* [21]. They are often designed a pair of primers to amplify the target gene; however, it is difficult to amplify all the sequences of *H. pylori* strains due to the large genetic differences and obvious genetic diversity of *H. pylori*, which might lead to false-negative results. The sensitivity of real-time PCR ranges from 63 to 85% [24–26]. We designed two pairs of primers for the conserved target gene glmM, which was the most sensitive and stable gene compared with the other genes, such as rpoB, 16SrRNA and ureA. Compared with a pair of primers, the method had a higher detection rate (100%) and better accuracy (*p* = 0.000). The method reduced the detection limit with a minimum of 6 bacterial copies per μL and increased the sensitivity to 100%. The AUC and Youden index were 0.988 and 0.98, respectively, which also showed the excellent detection performance of the method. Compared with the common clinical detection method RUT, quantitative RT-PCR showed better consistency with the classic gold standard of bacterial culture. However, the consistency degree was intermediate (0.41 < Kappa = 0.440 < 0.60) [27] because of the low bacterial culture rate (16.4%) due to the microaerobic growth characteristics of *H. pylori* strains. PCR was also a convenient method for detecting the cytotoxin-associated genes of *H. pylori*. In previous studies, the vacA, cagA and babA2 genes were tested successfully [18]. However, different primer sequences and reaction conditions were designed to run the PCR many times, which might lead to low throughput and extensive time consumption, so it is difficult to use in clinical practice. We designed a new real-time PCR system that could simultaneously test vacA s1, vacA m1, cagA and babA2. It took only 2 to 3 h from sample processing to test completion and had a good detective capacity (AUC: vacA s1 0.986, vacA m1 0.988, cagA 0.987, babA2 0.984; Youden index: vacA s10.94, vacA m10.95, cagA 0.9, babA2 0.9). The improvement of qPCR that makes it possible to be used in the clinic in the future.

*H. pylori* is an important human pathogen associated with most cases of peptic ulcer disease, gastritis and gastric cancer. One of the factors contributing to this phenomenon could be the patchy density of *H. pylori* that could be associated with chronic inflammation and activity [28]. Molnar et al. [29] found a significantly increased bacterial density in gastric erosions when compared with the healthy part of the gastric mucosa. Wang and Liu [30] reported that the relative amount of *H. pylori* infection in gastric cancer tissues was significantly higher than that in paracancerous normal tissues. These results confirmed the important relationship between the bacterial load and the relative diseases. Our results supported that the infectious amount of *H. pylori* was significantly positively associated with gastritis in corpus (r = 0.259, *p* = 0.003) and gastric erosion (r = 0.175, *p* = 0.043). Interestingly, we found that the amount of *H. pylori* infection in gastric precancerous patients was significantly lower than that in other *H. pylori*-positive patients (*p* = 0.021). Similar results were found in a study by Ladeira et al. [31]. Zhang et al. [32] proposed that atrophic mucosa and intestinal metaplasia are detrimental to the growth of *H. pylori*, and Tang et al. [33] indicated that the cancerous epithelium microenvironment and the changes experienced by cancer cells were detrimental to the survival of the bacteria. These findings suggested that a decrease in *H. pylori* did not necessarily indicate that you are safe from developing cancer but rather that you should undergo regular endoscopies and pay attention to the absolute number of bacteria in your stomach.

On the other hand, in addition to the density of bacteria, the presence of bacterial virulence factors, such as vacA, cagA and babA, at the epithelium would increase *H. pylori* colonization and the susceptibility to the associated diseases [19]. There have been few studies on the prevalence of *H. pylori* and its vacA, cagA and babA2 genotypes in the Chinese population. Our study showed vacA s1 to be predominant, with 53.3, 80 and 53.8% of patients carrying this marker in chronic superficial gastritis, peptic gastric ulcer and erosive gastritis, respectively (*p* < 0.05), which is similar to findings reported elsewhere, such as in South Africa [25], India [34] and Mexico [18]. Some studies mentioned that the prevalence rates of vacAs1 were significantly increased in peptic ulcer [25] and gastric cancer [18]. We did not find the same phenomenon in our studies. There were no significant differences in the prevalence of *H. pylori*-vacA s1+/vacA m1+/cagA+/babA2+ in the different gastritis diseases. Similar findings regarding cagA/babA2 were made in Mexican patients [18], and vacA/cagA had no correlation with pathology in a Nigerian study [35]. Our results suggested that vacA s1 played more important roles in inducing chronic superficial gastritis, peptic ulcer and erosive gastritis than vacA m1, cagA and babA2, which might be partly due to the amount of *H. pylori* significantly increasing when patients were infected with vacA s1+ strains (*p* = 0.025). Although virulence factors and bacterial load are different aspects describing infection, some virulence factors could indeed cause an increase in bacterial load [36]. The specific mechanism needs to be further studied. Meanwhile, the frequency of *H. pylori*-vacA+/cagA+/babA2+ strains showed a significant positive correlation. In other words, they often appeared together in the same patients, especially vacAs1, cagA and babA2 (*p* < 0.01). This finding was in accordance with previous reports. vacA s1 was often linked with the presence of the cagA/babA2 genotype, and babA2 status was significantly associated with cagA genotype-positive strains [12, 13, 20, 37, 38]. Therefore, neither of the virulence markers could be considered an independent factor for disease outcome [39]. In fact, when multiple virulence factors were present, the risk of a severe clinical outcome was elevated [40]. In conclusion, the amount of *H. pylori* infection and the *H. pylori* virulence factors showed complex correlations with the occurrence and development of gastric diseases. We should proceed with precision examinations of the infected patients in the clinic to analyse and propose reasonable treatment strategies. All of these findings could be well implemented in the future, along with the improvement in *H. pylori* detection technology by qPCR.

## Conclusions

The *H. pylori* infectious amount and presence of *H. pylori* virulence factors showed complex correlations with the occurrence and development of gastric diseases. We improved the qPCR method, including designing two pairs of primers and probes to target the glmM gene and establishing a multiplex qPCR method for virulence factor detection. The method with good detection performance could detect the *H. pylori* infectious amount quickly and accurately and test vacA (s1/m1), cagA and babA2 status of *H. pylori* simultaneously; thus, it could be used for precision detection of *H. pylori.* The improved method was used to test *H. pylori* in clinical patients and provided valuable information for disease diagnosis and treatment. First, some patients with a high number of *H. pylori* copies would clearly benefit from antibiotic treatment. Second, these patients should be treated with bacterial eradication therapy as soon as possible, especially those infected with strains carrying the virulence vacA s1 gene or multiple virulence genes simultaneously. Last, we suggest that doctors should focus on patients who have been infected with *H. pylori* in the past but exhibit significantly reduced numbers of bacteria without any treatment. They may have the potential to suffer cancerous development.

## Methods

### Patients and specimens

A total of 141 consecutive patients (83 women and 58 men with an age of 50.94 ± 12.24 years) who underwent upper endoscopy due to dyspeptic symptoms at Xinqiao Hospital, Chongqing, China, during October 2017 and November 2017 were recruited. A rapid urease test (RUT), *H. pylori* culturing and qPCR were performed on specimens. A patient was considered positive for *H. pylori* infection when at least two of these three tests gave positive results. A negative *H. pylori* infection status was considered if two of the three tests performed gave concordant negative results.

### RUT and culturing of H. pylori

Two biopsy specimens were taken from different places in the stomach of one patient according to the clinical practice to avoid bleeding. One was sent for RUT, and the other was divided into two pieces for culturing and qPCR.

The RUT was performed using *H. pylori* rapid urease test paper (Zhuhai Kedi Technology Co., Ltd., 151,102) and read within 30 s for all cases. To culture *H. pylori*, the biopsy specimens were applied to the surfaces of self-made blood plates (200 mL of Skirrow medium, 3.5 g of agar powder, 10 mL of 10% glucose, 10 mL of defibrated sheep blood and 1 mL of composite antibiotic). The inoculated plates were placed in a vertical culture bag (Mitsubishi Gas Chemical Company, Inc. MGC, C-43) together with an AnaeroPack (Mitsubishi Gas Chemical Company, Inc. MGC, C-2) to generate a microaerophilic environment (5% O_2_ concentration; 10% CO_2_ concentration; 85% N_2_ concentration) and incubated for 3 to 5 days. *H. pylori* microorganisms were identified on the basis of characteristic colony morphology, typical appearance on Gram staining, positive urease and catalase tests, PCR and sequencing.

### The process of qPCR

#### DNA extraction

TRIzol reagent (bioPerfectus technologies, SDK60103) was used to extract total DNA from gastric biopsy sections.

#### Detection of H. pylori

The gene glmM was used as a target gene, as it was the most sensitive and stable gene compared with the other genes, such as rpoB, 16SrRNA and ureA (Supplementary Table 5). The sequences of the genes encoding glmM of *H. pylori* was searched from GenBank. One primer and one probe were designed based on the normal gene sequences. The other primer was designed based on the mutation of T187C and A189C in glmM, and the other probe was designed based on the mutation of C231T in glmM. Primers and probes were designed using NCBI (https://www.ncbi.nlm.nih.gov/), and synthesized by bioPerfectus Technologies (Shanghai). The sequences of the primers and probes are shown in Table 5. The qPCR system was prepared according to the instructions, and a total of 25 μL was prepared: 12.5 μL of 2 × Probe qPCR Mix, 8 μL of primer and probe mix and 4.5 μL of template DNA. The qPCR Mix (12.5 μL) included FastStart™ Taq DNA Polymerase (Roche 12,032,937,001, 5 U/ul, 0.2 μL), GeneAmp™ PCR Buffer II & MgCl_2_ (Applied Biosystems™ 4,379,878, 5×, 5 μL), MgCl_2_ (Thermo Scientific™, AB0359, 25 Mm, 3 μL), dUTP (Thermo Scientific™, R0133, 100 mM, 0.0625 μL), dTTP (Invitrogen™, 10,219,012, 100 mM, 0.0625 μL), dATP (Invitrogen™, 10,216,018, 100 mM, 0.125 μL), dGTP (Invitrogen™, 10,218,014, 100 mM, 125 μL), dCTP (Invitrogen™, 10,217,016, 100 mM, 0.125 μL), AmpErase™ Uracil N-Glycosylase (UNG) (Applied Biosystems™, N8080096, 2 U/μL, 0.1 μL) and DNase/RNase-free ddH_2_O (Solarbio, R1600, 3.7 μL). The primer and probe mix (400 μL/50 samples) included F1 (100 μM) 3.75 μL, F2 (100 μM) 3.75 μL, R (100 μM) 7.5 μL, P1 (100 μM) 2.5 μL, P2 (100 μM) 2.5 μL and 380 μL of double distilled water. Using a Bio-Rad quantitative real-time fluorescence PCR instrument (CFX96) for PCR amplification, the reaction conditions were as follows: 95 °C for 3 min followed by 95 °C for 10 s and 58 °C for 40 s for a total of 45 cycles. The PCR product was stored at 4 °C. The average value of the experiment was obtained from the assay repeated three times.

**Table 5:** glmM primers and probes sequences

Category	Sequence (5′ → 3′)
Forward primer 1(F1)	GCTCTCACTTCCATAGGCTATAATG
Forward primer 2(F2)	GCTTTAACTTCCATAGGCTATAATGT
Reverse primer(R)	GCGCATGTCTTCGGTTAAAA
Probe 1(P1)	FAM-TAGGGCCTATGCCTACCCCTGCGA-HBQ1^a^
Probe 2 (P2)	FAM-TAGGGCCTATGCCCACCCCTGC-HBQ1

^a^FAM is the fluorescence reporter, and HBQ1 is the fluorescence quencher

#### Quantifying H. pylori

The detailed quantitative steps are described in the supplementary files. In short, we used house-keeper gene GAPDH as an internal control. First, the plasmid PUC57-GAPDH which contains the GAPDH gene (Synthesized by Shanghai Sangon Biotech) was quantified by qPCR with the Human DNA Quantitation Standard (NCBI, SRM2372) as the reference standards, and the copy number of GAPDH on the plasmid which was equal to that of AMP^r^ contained in PUC57 was calculated. Second, the different concentrations of the plasmid PUC57-GAPDH with a known copy number were quantified by qPCR with the primer and probe of AMP^r^ to obtain the quantitative formula. The formula was used to calculate the number of copies of PUC57-glmM (Synthesized by Shanghai Sangon Biotech), which was quantified by qPCR with the primer and probe of AMP^r^. Last, the plasmid PUC57- glmM with a known copy number was quantified by qPCR with the primer and probe of glmM. According to the test result, the following calculation formula could be obtained: Ct = − 3.4 × log (copies/μL) + 37.18 (R^2^ > 0.99). This method could be used to quantify other samples (Supplementary Tables 6–11).

#### Detection of the virulence factors of H. pylori

GenBank was searched for the sequences of the genes encoding vacA s1, vacA m1, cagA and babA2 of *H. pylori*. The probes of vacA s1 were designed based on the two genotypes of vacA s1 (vacA s1a and vacA s1b). The forward primer2 of vacA m1 were designed based on the mutation of T2037C and T2041C, and the probe2 of vacA m1 was designed based on the mutation of T2115C, T2124C and T2127A. The primer and probe of cagA were designed based on the normal gene sequences. The probe2 of babA2 was designed based on the mutation of A283G and A286G. Primers and probes were designed by using NCBI (https://www.ncbi.nlm.nih.gov/). Primers and probes were synthesized by Sangon Biotech (Shanghai). The sequences of primers and probes are shown in Table 6. The real-time PCR system was prepared according to the instructions, and a total of 25 μL was prepared: 12.5 μL of 2 × Probe qPCR Mix, 8 μL of primer and probe mix and 4.5 μL of template DNA. The primer and probe mix (400 μL/50 samples) included vacA s1: F (100 μM) 7.5 μL, R (100 μM) 7.5 μL, P1 (100 μM) 5 μL, P2 (100 μM) 5 μL; vacA m1: F1 (100 μM) 3.75 μL, F2 (100 μM) 3.75 μL, R (100 μM) 7.5 μL, P1 (100 μM) 5 μL, P2 (100 μM) 5 μL; cagA: F (100 μM) 7.5 μL, R (100 μM) 7.5 μL, P(100 μM) 5 μL; babA2: F (100 μM) 7.5 μL, R (100 μM) 7.5 μL, P1 (100 μM) 5 μL, P2 (100 μM) 5 μL and 305 μL of double distilled water. Using the Bio-Rad quantitative real-time fluorescence PCR instrument (CFX96) for PCR amplification, the reaction conditions were as follows: 95 °C for 3 min, followed by 95 °C for 10 s and 51 °C for 40 s for a total of 40 cycles. The PCR product was stored at 4 °C. The average value of the experiment was obtained from the assay repeated three times.

**Table 6:** The primers and probe sequences for the virulence factors

Virulence Factor	Category	Sequence (5′ → 3′)^**a**^
vacA s1	Forward primer (F)	ACACCGCAAAATCAATCGCC
	Reverse primer (R)	CCCCAACAATGGCTGGAATG
	Probe 1 (P1)	HEX-GCATCACACCGCAACAAAGT-BHQ2
	Probe 2 (P2)	HEX-GCGCCATACCGCAAGAGAGT-BHQ2
vacA m1	Forward primer 1 (F1)	ATCAATTATTTGGTCCGAGGC
	Forward primer 2 (F2)	ATCAACTATCTGGTCCGAGGC
	Reverse primer (R)	GCTGTTAATCTTCATGAGCGGT
	Probe 1 (P1)	FAM-GATAGCGCGACTGGGTTTTA-BHQ1
	Probe 2 (P2)	FAM-GACAGCGCGACCGGATTTTA-BHQ1
cagA	Forward primer (F)	GTGCCTGCTAGTTTGTCAGC
	Reverse primer (R)	ACGAGCTTAAGCCACTCAGG
	Probe 1 (P)	Texas Red-GCTATTAACAGCCACACACGC-Dabcyl
babA2	Forward primer (F)	GAAATCCCTAATACTAAATCC
	Reverse primer (R)	GTAAAAGCCGTCGTCTTCAGC
	Probe 1 (P1)	CY5-GGAGAAAAAACATGAAAAAACA-BHQ3
	Probe 2 (P2)	CY5-GGGGAGAAAACATGAAAAAACA-BHQ3

^a^Note: HEX, FAM, Texas Red and CY5 are the fluorescence reporters. BHQ1, BHQ2, BHQ3 and Dabcyl are the fluorescence quenchers

### Statistical analyses

SPSS13.0 for Windows (SPSS, Inc., Chicago, IL) was used for statistical analysis of data. Measurement data were analysed by t-test and χ^2^ test. The t-test was expressed as the mean ± standard deviation. Kappa and McNemar tests were used to compare consistency between different *H. pylori* detection methods. Product-moment correlation was used to analyse the relationship between different variables. *P* < 0.05 was considered statistically significant.

## Supplementary information

**Additional file 1: Supplementary Fig. 1.** Comparison of copy number between two primer design strategies. **Supplementary Table 1.** The sequences of a pair of primers and probe for glmM. **Supplementary Table 2.** Performance of quantitative RT-PCR testing *H. pylori.***Supplementary Table 3.** Determination of *H. pylori* infection status in 141 patients. **Supplementary Table 4.***H. pylori* virulence factor detection in patients. **Supplementary Table 5.** The detection results of glmM, rpoB, 16SrRNA and ureA. **Supplementary Table 6.** GAPDH primers and probe sequences (138 bp). **Supplementary Table 7.** The quantitative results of PUC57-GAPDH. **Supplementary Table 8.** AMP^r^ primers and probe sequences (110 bp). **Supplementary Table 9.** The quantitative results of AMP^r^. **Supplementary Table 10.** The quantitative results of PUC57-glmM. **Supplementary Table 11.** The quantitative results of glmM.

## Data Availability

All data involved in this study is available upon reasonable request to the corresponding author.

## Abbreviations

*H. pylori*: *Helicobacter pylori*
RUT: Rapid urease test
VacA: Vacuolating cytotoxin A
CagA: Cytotoxin-associated gene A
BabA: Blood group antigen-binding adhesion
PCR: Polymerase chain reaction
qPCR: Quantitative real-time polymerase chain reaction
LOD: Limit of detection
AUC: Area under the ROC curve
CFU: Colony-forming unit
NPV: Negative predictive values
PPV: Positive predictive values
