# Supplementary material for: An improved quantitative real-time polymerase chain reaction technology for Helicobacter pylori detection in stomach tissue and its application value in clinical precision testing
Source: BMC Biotechnol. 2020 Jun 22;20:33. doi: 10.1186/s12896-020-00624-z (PMC7310109; doi:10.1186/s12896-020-00624-z)
Supplement: Supplementary file 1 — Additional file 1: Supplementary Fig. 1. Comparison of copy number between two primer design strategies. Supplementary Table 1. The sequences of a pair of primers and probe for glmM. Supplementary Table 2. Performance of quantitative RT-PCR testing H. pylori.Supplementary Table 3. Determination of H. pylori infection status in 141 patients. Supplementary Table 4.H. pylori virulence factor detection in patients. Supplementary Table 5. The detection results of glmM, rpoB, 16SrRNA and ureA. Supplementary Table 6. GAPDH primers and probe sequences (138 bp). Supplementary Table 7. The quantitative results of PUC57-GAPDH. Supplementary Table 8. AMPr primers and probe sequences (110 bp). Supplementary Table 9. The quantitative results of AMPr. Supplementary Table 10. The quantitative results of PUC57-glmM. Supplementary Table 11. The quantitative results of glmM. [file 12896_2020_624_MOESM1_ESM.docx]

**Supplementary Files**

*Comparison of different primers*

Twenty samples with 6 copies/μL of *H. pylori* (26695) mixed with normal gastric tissues were prepared. TRIzol reagent (bioPerfectus technologies, SDK60103) was used to extract total DNA from gastric biopsy sections. Primers and probes were designed using NCBI (<https://www.ncbi.nlm.nih.gov/>), and synthesized by bioPerfectus technologies (Shanghai). The sequences of the primers and probes are shown in Supplementary Table 1. The qPCR system was prepared according to the instructions, and a total of 25 μL was prepared: 12.5 μL of 2×Probe qPCR Mix, 8 μL of primer and probe mix and 4.5 μL of template DNA. The primer and probe mix (400 μL/50 samples) included F1 (100 µM) 7.5 μL, R (100 µM) 7.5 μL, P1 (100 µM) 5 μL and 380 μL of double-distilled water. Using a Bio-Rad quantitative real-time fluorescence PCR instrument (CFX96) for PCR amplification, the reaction conditions were as follows: 95°C for 3 min followed by 95°C for 10 s and 58°C for 40 s for a total of 45 cycles. The PCR product was stored at 4°C. The average value of the experiment was obtained from the assay repeated three times. The process of qPCR using two pairs of primers was described in the manuscript. The plasmid PUC57- glmM with a known copy number was quantified by qPCR with the primers and probe of glmM (Supplementary Table 9). According to the test result, the calculation formula could be obtained: Ct=-3.4×log (copies/μL) +37.18 (R^2^>0.99) which was used to quantify other samples. The results showed that 17 out of 20 samples tested with a pair of primers failed to detect positive results, thus the detection rate was 15%. The detection rate of two pairs of primers in 20 samples was 100%. There was a significant difference in copy numbers between the two types of primers (Supplementary Figure1).

**Supplementary Table** **1**. The sequences of a pair of primers and probe for glmM.

| **Category** | **Sequence (5’→3’)** |
| --- | --- |
| Forward primer 1 (F1) | GCTCTCACTTCCATAGGCTATAATG |
| Reverse primer (R) | GCGCATGTCTTCGGTTAAAA |
| Probe1 (P1) | FAM-TAGGGCCTATGCCTACCCCTGCGA-HBQ1a |

**Supplementary Figure 1.** Comparison of copy number between two primer design strategies


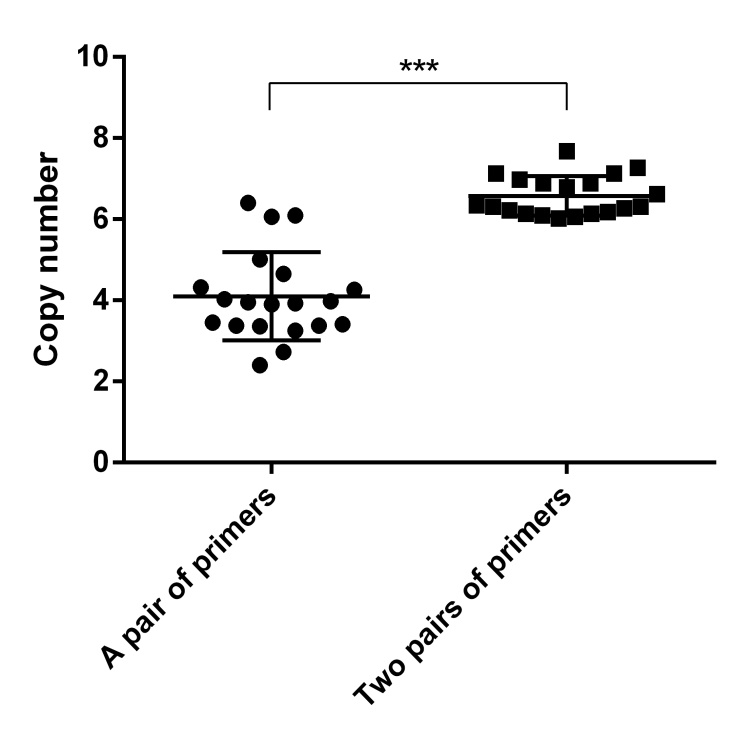


**Supplementary Table 2.** Performance of quantitative RT-PCR testing *H. pylori.*

| **No. of positive**  **specimens** | **No. of negative**  **specimens** | **No. of false-positive**  **specimens** | **No. of false-negative**  **specimens** | **Sensitivity**  **(%)** | **Specificity**  **(%)** | **PPV (%)** | **NPV (%)** |
| --- | --- | --- | --- | --- | --- | --- | --- |
| 22 | 79 | 33 | 0 | 100 | 70.5 | 40 | 100 |

**Supplementary Table 3.** Determination of *H. pylori* infection status in 141 patients.

| **Group no.** | **No. of patients** | **Result by:** | | | ***H. pylori***  **infection status** |
| --- | --- | --- | --- | --- | --- |
|  |  | **RUT** | **RT-PCR** | **Culture** |  |
| Ⅰ | 20 | Positive | Positive | Positive | Positive |
| Ⅱ | 1 | Negative | Positive | Positive | Positive |
| Ⅲ | 1 | ND^a^ | Positive | Positive | Positive |
| Ⅳ | 31 | Positive | Positive | Negative | Positive |
| Ⅴ | 17 | Negative | Negative | Negative | Negative |
| Ⅵ | 2 | Negative | Positive | Negative | Negative |
| Ⅶ | 56 | Positive | Negative | Negative | Negative |
| Ⅷ | 6 | ND | Negative | Negative | Negative |
| Ⅸ | 7 | ND | Positive | ND | Excluded |

^a^ND, not done.

**Supplementary Table** **4.** *H. pylori* virulence factor detection in patients.

| **Patient no.** | **vacA** | | **cagA** | **babA2** | **Patient no.** | **vacA** | | **cagA** | **babA2** |
| --- | --- | --- | --- | --- | --- | --- | --- | --- | --- |
|  | **m1** | **s1** |  |  |  | **m1** | **s1** |  |  |
| 1 | ﹣ | + | ﹣ | ﹣ | 50 | ﹣ | + | + | ﹣ |
| 3 | ﹣ | ﹣ | ﹣ | ﹣ | 51 | ﹣ | ﹣ | ﹣ | ﹣ |
| 4 | ﹣ | + | ﹣ | + | 52 | ﹣ | ﹣ | ﹣ | ﹣ |
| 5 | ﹣ | + | ﹣ | ﹣ | 54 | + | + | + | ﹣ |
| 6 | + | + | + | + | 56 | ﹣ | ﹣ | ﹣ | ﹣ |
| 9 | ﹣ | + | + | + | 60 | ﹣ | ﹣ | ﹣ | ﹣ |
| 13 | ﹣ | + | ﹣ | ﹣ | 61 | + | + | + | + |
| 14 | ﹣ | + | ﹣ | + | 63 | ﹣ | ﹣ | ﹣ | ﹣ |
| 17 | ﹣ | ﹣ | ﹣ | ﹣ | 65 | ﹣ | ﹣ | ﹣ | ﹣ |
| 19 | ﹣ | ﹣ | ﹣ | + | 67 | ﹣ | ﹣ | ﹣ | ﹣ |
| 20 | + | + | ﹣ | ﹣ | 71 | ﹣ | + | ﹣ | ﹣ |
| 23 | ﹣ | ﹣ | ﹣ | ﹣ | 72 | ﹣ | ﹣ | ﹣ | ﹣ |
| 24 | ﹣ | + | ﹣ | + | 80 | ﹣ | + | + | + |
| 25 | ﹣ | ﹣ | ﹣ | ﹣ | 82 | ﹣ | ﹣ | ﹣ | ﹣ |
| 27 | ﹣ | + | ﹣ | ﹣ | 83 | ﹣ | ﹣ | ﹣ | ﹣ |
| 29 | ﹣ | ﹣ | ﹣ | ﹣ | 84 | ﹣ | + | + | ﹣ |
| 32 | ﹣ | + | ﹣ | + | 86 | ﹣ | ﹣ | ﹣ | ﹣ |
| 33 | + | + | + | + | 88 | ﹣ | ﹣ | ﹣ | ﹣ |
| 35 | ﹣ | ﹣ | ﹣ | ﹣ | 89 | ﹣ | + | ﹣ | ﹣ |
| 36 | ﹣ | ﹣ | ﹣ | ﹣ | 94 | ﹣ | ﹣ | ﹣ | ﹣ |
| 37 | ﹣ | + | + | + | 95 | ﹣ | ﹣ | ﹣ | ﹣ |
| 38 | ﹣ | + | + | ﹣ | 111 | + | + | + | + |
| 40 | ﹣ | + | ﹣ | ﹣ | 112 | ﹣ | + | + | ﹣ |
| 43 | ﹣ | + | + | + | 120 | + | + | ﹣ | ﹣ |
| 45 | ﹣ | + | ﹣ | ﹣ | 133 | ﹣ | + | ﹣ | ﹣ |
| 48 | ﹣ | ﹣ | ﹣ | ﹣ | 138 | ﹣ | + | ﹣ | + |
| 49 | ﹣ | ﹣ | + | ﹣ |  |  |  |  |  |
| Total | 3 | 17 | 7 | 10 |  | 4 | 12 | 7 | 4 |

*Identification of target gene*

We detected the same sample using glmM, rpoB, 16SrRNA and ureA as the target gene. The results showed that the Ct value of glmM was 1.81, 1.88 and 1.2 lower than that of rpoB, 16SrRNA and ureA, respectively, and the Cv value was the smallest among these (Supplementary Table 5).

**Supplementary Table 5.** The detection results of glmM, rpoB, 16SrRNA and ureA.

| **The target gene** | **Ct1** | **Ct2** | **Ct3** | **The mean Ct** | **Cv** |
| --- | --- | --- | --- | --- | --- |
| glmM | 19.54 | 19.96 | 19.78 | 19.76 | 1.06% |
| rpoB | 20.77 | 22.07 | 21.88 | 21.57 | 3.25% |
| 16SrRNA | 21.01 | 21.91 | 22.01 | 21.64 | 2.54% |
| ureA | 20.08 | 21.53 | 21.28 | 20.96 | 3.70% |

*Quantification of H. pylori*

According to the instructions, the Human DNA Quantitation Standard (NCBI, SRM2372), which contained GAPDH, was diluted to 50 ng/μL, 40 ng/μL, 25 ng/μL, 10 ng/μL and 1 ng/μL. The plasmid PUC57-GAPDH (Synthesized by Shanghai Sangon Biotech) was quantified by qPCR with the primers and probe of GAPDH (Supplementary Table 6-7). The software Light Cycler 480 was used to calculate the copy number of GAPDH on the plasmid, and 1.14×10^9^ copies/μL were obtained from the mean of the PUC57-GAPDH 10^-5^ and PUC57-GAPDH 0.5×10^-5^ copies based on the following formula: Ct=-3.52×log(copies/μL)+35.49(R^2^>0.99). (Note: 1 ng of The Human DNA Quantitation Standard is approximately 290×2 copies GAPDH).

**Supplementary Table 6.** GAPDH primers and probe sequences (138 bp).

| **Category** | **Sequence (5’→3’)** |
| --- | --- |
| Forward primer (F) | GAAGGTGAAGGTCGGAGTCAA |
| Reverse primer (R) | TTCCCGTTCTCAGCCATGTAGT |
| Probe (P) | GGATTTGGTCGTATTGGGCG |

**Supplementary Table 7.** The quantitative results of PUC57-GAPDH.

| **The sample dilution multiple** | **The mean Ct** | **The copy number** |
| --- | --- | --- |
| 50ng | 19.755 | 29000 |
| 40ng | 20.11 | 23200 |
| 25ng | 20.79 | 14500 |
| 10ng | 22.33 | 5800 |
| 1ng | 25.725 | 580 |
| PUC57-GAPDH 10^-5^ | 21.19 | 11547 |
| PUC57-GAPDH 0.5×10^-5^ | 22.3 | 5586 |

The copy number of GAPDH on the plasmid was equal to that of AMP^r^ contained in PUC57. Then, the different concentrations of the plasmid PUC57-GAPDH with a known copy number was quantified by qPCR with the primers and probe of AMP^r^ (Supplementary Tables 8-9). The quantitative calculation formula Ct=-3.27×log(copies/μL)+35.18 (R^2^>0.99) was obtained by the software Light Cycler 480.

**Supplementary Table 8.** AMP^r^ primers and probe sequences (110 bp).

| **Category** | **Sequence (5’→3’)** |
| --- | --- |
| Forward primer (F) | ACCCAGAAACGCTGGTGAAA |
| Reverse primer (R) | GGGGCGAAAACTCTCAAGGA |
| Probe (P) | AGATCAGTTGGGTGCACGAG |

**Supplementary Table 9.** The quantitative results of AMP^r^.

| **The sample dilution multiple** | **The mean Ct** | **The copy number** |
| --- | --- | --- |
| PUC57-GAPDH 10^-3^ | 15.18 | 1140000 |
| PUC57-GAPDH 10^-4^ | 18.39 | 114000 |
| PUC57-GAPDH 0.5×10^-4^ | 20.02 | 57000 |
| PUC57-GAPDH 10^-5^ | 21.71 | 11400 |
| PUC57-GAPDH 0.5×10^-5^ | 23.34 | 5700 |
| PUC57-GAPDH 10^-6^ | 25.01 | 1140 |
| PUC57-GAPDH 10^-7^ | 28.32 | 114 |

The plasmid PUC57-glmM (Synthesized by Shanghai Sangon Biotech) was quantified by qPCR with the primers and probe of AMP^r^ (Supplementary Table 10). The number of copies of PUC57-glmM could be calculated according to the formula Ct=-3.27×log(copies/μL)+35.18 (R^2^>0.99). The PUC57-glmM result was 2.42×10^9^ copies/μL from the mean of PUC-57-glmm 10^-5^ and PUC-57-glmm 10^-6^ copies.

**Supplementary Table 10.** The quantitative results of PUC57-glmM.

| **The sample dilution multiple** | **The mean Ct** | **The copy number** |
| --- | --- | --- |
| PUC-57-glmm 10^-5^ | 20.82 | 24628 |
| PUC-57-glmm 10^-6^ | 24.14 | 2377 |

The plasmid PUC57-glmM with a known copy number was quantified by qPCR with the primers and probe of glmM (Supplementary Table 11). According to the test result, the calculation formula could be obtained: Ct=-3.4×log (copies/μL) +37.18 (R^2^>0.99). This could be used to quantify other samples.

**Supplementary Table 11.** The quantitative results of glmM.

| **The copy number of PUC57- glmM** | **The mean Ct** |
| --- | --- |
| 2.42×10^-1^ | N/A |
| 2.42×10^0^/4 | N/A |
| 2.42×10^0^/2 | 39.15 |
| 2.42×10^0^ | 35.92 |
| 2.42×10^1^ | 32.61 |
| 2.42×10^2^ | 28.90 |
| 2.42×10^3^ | 25.59 |
| 2.42×10^4^ | 22.21 |
| 2.42×10^5^ | 18.77 |
